# Platelet Glycoprotein Receptor Ia-C807T and IIIa-PlA1/PlA2 Genetic Polymorphisms Are Associated With Enhanced Platelet Function in Women With Recurrent Miscarriages

**DOI:** 10.7759/cureus.47832

**Published:** 2023-10-27

**Authors:** Vassilios Tsamadias, Nikolaos Vlachadis, Styliani Demeridou, Evaggelia Kouskouni, George Creatsas, Nikolaos F Vlahos, Emmanuel Economou

**Affiliations:** 1 Clinical Laboratory for Therapeutic Individualization, Second Department of Obstetrics and Gynecology, National and Kapodistrian University of Athens Medical School, Aretaieio University Hospital, Athens, GRC; 2 Department of Biopathology - Microbiology and Biochemistry, National and Kapodistrian University of Athens Medical School, Aretaieio University Hospital, Athens, GRC; 3 Second Department of Obstetrics and Gynecology, National and Kapodistrian University of Athens Medical School, Aretaieio University Hospital, Athens, GRC

**Keywords:** platelet function, genetic polymorphisms, spontaneous abortions, miscarriages, pfa-100, platelets

## Abstract

Introduction: Thrombophilic genetic polymorphisms of the platelet glycoproteins Ia (GpIa) and IIIa (GpIIIa) have been associated with an increased risk of recurrent miscarriages. The aim of this study was to investigate the association of genetic polymorphisms GpIa-C807T and GpIIIa-T1565C-PlA1/PlA2 with platelet function in women with unexplained spontaneous recurrent miscarriages.

Methods: This cross-sectional study comprised 196 unrelated nulliparous Greek women with a history of unexplained recurrent miscarriages. Patients were genotyped for the presence of the GpIa-C807T (rs1126643) and GpIIIa-T1565C-PlA1/PlA2 (rs5918) genetic polymorphisms by pyrosequencing, and the collagen/epinephrine closure time (COL/EPI CT) of the subjects was assessed using the platelet function analyzer (PFA)-100.

Results: In the total population of women with recurrent miscarriages, the COL/EPI CT ranged from 70 to 160 seconds (median: 122 seconds, interquartile range (IQR): 102.3-138 seconds). In comparison with the double homozygotes CC/PlA1PlA1 that had the most prolonged CT (mean: 131.9 ± 17.5 seconds), the COL/EPI CT was statistically significantly shorter for the GpIa-807T single carriers (mean: 120.3 ± 20.9 seconds) (p=0.011) (absolute difference: 11.6 seconds, 95% confidence interval (CI): 21.2 to -2.0 seconds; relative difference: -9%, 95% CI: -16% to -2%), and the GpIIIa-PlA2 single carriers also displayed a trend for shorter COL/EPI CT (mean: 121.3 ± 23.7 seconds) (p=0.141) (absolute difference: -10.6 seconds, relative difference: -8%), whereas the combined carriers of the GpIa-807T and the GpIIIa-PlA2 alleles exhibited the shortest COL/EPI CT (mean: 104.1 ± 19.7 seconds) (absolute difference: -27.7 seconds, 95% CI: -39.1 to -16.3 seconds; relative difference: -21%, 95% CI: -30% to -12%) (p<0.001). In comparing genotype frequencies in the lower half with those in the upper half of the COL/EPI CT range, the GpIa-807T and the GpIIIa-PlA2 single carriers were associated with higher odds of COL/EPI CT < 122 seconds (odds ratio (OR)=3.4, 95% CI: 1.5 to 7.5, p=0.002, and OR=2.6, 95% CI: 1.0 to 7.2, p=0.053, respectively). The association was strongest for the combined carriers with OR of 15.0 (95% CI: 5.2 to 43.2, p<0.001) for COL/EPI CT below the median and OR of 35.5 (95% CI: 4.4 to 284.5, p<0.001) for COL/EPI CT < 100 seconds.

Conclusion: The GpIa-C807T and GpIIIa-PlA1/PlA2 polymorphisms and more pronouncedly the combined carriers of the risk variants are associated with enhanced platelet reactivity expressed via shorter COL/EPI CT. These findings provide further evidence for the role of platelet-associated genetic thrombophilia in the pathogenesis of recurrent miscarriages and promote the analysis of platelet function as a diagnostic tool in the evaluation of this disorder.

## Introduction

Miscarriage is defined as fetal loss during the first two trimesters of pregnancy, and they are the most common serious complications in the first half of pregnancy. Overall, miscarriages after clinically diagnosed pregnancies occur at a rate of 15%-20%. Recurrent miscarriages are defined as the occurrence of at least two miscarriages in the same woman. It appears that they constitute a separate syndrome requiring a special diagnostic and therapeutic approach and occur in up to 5% of women. The main cause of miscarriages is chromosomal abnormalities; however, a variety of other factors have been associated with an increased risk of pregnancy loss, including anatomical abnormalities of the uterus, infections, sexually transmitted diseases, and immunological and hormonal disorders. Of note, in approximately 50% of cases, the etiology remains unexplained [1-5].

Over the past 2-3 decades, numerous studies have reported on the role of genetic polymorphisms in the etiopathogenesis of recurrent miscarriages, with particular emphasis on the role of genetic thrombophilia [6-8]. Possible biological mechanisms explaining the role of genetic thrombophilia in the incidence of miscarriage include impaired blood supply to the fetus in early pregnancy and abnormal placentation [9]. Although the majority of hereditary thrombophilic factors that have emerged involve coagulation factors, such as Leiden factor V G1691A and the prothrombin G20210A mutation [8-10], we have previously reported on the role of platelet-derived thrombophilia in spontaneous miscarriages, and in particular, we found that the genetic polymorphisms of the platelet glycoproteins Ia (GpIa-C807T) and IIIa (GpIIIa-T1565C-PlA1/PlA2) are associated with increased risk of spontaneous recurrent miscarriages. These genetic variants have been associated with increased platelet adhesion to collagen and activation through the GpIa-IIa receptor and amplified platelet aggregation by augmenting the binding activity of the GpIIb-IIIa receptor to fibrinogen [11].

The platelet function analyzer (PFA)-100 is a rapid, simple, and reproducible test of platelet function through the assessment of primary hemostasis in vitro. It is based on platelet adhesion, activation, and aggregation under high shear stress conditions. The platelet function is determined by the time required to close a microscopic aperture cut in a membrane with platelet aggregates. This time is expressed as closure time (CT), and the membrane is coated by collagen and epinephrine (COL/EPI) [12].

In the present study, we aimed to examine platelet function using the PFA-100 method and investigate the possible associations with the GpIa-C807T and GpIIIa-PlA1/PlA2 genetic polymorphisms in a group of women with unexplained recurrent spontaneous abortions.

## Materials and methods

This was a cross-sectional study conducted in the Clinical Laboratory for Therapeutic Individualization at the Second Department of Obstetrics and Gynecology, Aretaieio University Hospital, Athens, Greece. The study was approved by the Ethics Committee of the Aretaieio University Hospital. Informed consent was provided by all participants, and the procedures were in compliance with the Declaration of Helsinki regarding research involving human subjects.

A total of 196 unrelated nulliparous Greek women aged up to 40 years with at least two spontaneous miscarriages after spontaneous conception and before 20 weeks of gestation were included in this study. The mean number of miscarriages in the group of patients was 2.6 (± 0.8). All cases had negative results after routine testing for typical risk factors for miscarriages, by ultrasound, hysteroscopy, or hysterosalpingogram for anatomical deformities, and by appropriate laboratory tests for the detection of relevant microbiological, hormonal, and immunological disorders. They had a normal karyotype, as well as their partners, and did not take any medication. Further exclusion criteria included hematocrit values <35% or >45% and platelet counts <100 × 10^9^.

Patients were genotyped for the presence of the GpIa-C807T (rs1126643) and GpIIIa-T1565C-PlA1/PlA2 (rs5918) genetic polymorphisms, and then, the platelet function of the women was assessed by PFA-100 by determining the closure time (CT) with collagen and epinephrine used as an agonist (COL/EPI).

Pyrosequencing was used for genotyping as previously described [11]. In short, the genomic DNA was extracted from peripheral blood, and PyroMark Assay Design 2.0 software (Qiagen, Hilden, Germany) was used to design polymerase chain reaction (PCR) primers (GpIa: forward 5΄-CAG CCC ATT AAT AAA TGT CTC CTC-3', reverse 5΄-AGC ACC AAA ACT TAC CTT GCA TAT-3΄-biotinylated; band size: 196 bp, GpIIIa: forward 5΄-TGC TCC AAT GTA CGG GGT AA-3΄, reverse 5΄-CCT CAC TCA CTG GGA ACT CGA-3΄-biotinylated; band size: 208 bp) and pyrosequencing primers (GpIa: 5΄-GGG GAC CTC ACA AAC A-3΄, GpIIIa: 5΄-TTG GGC TCC TGT CTT Α-3΄). PCR was carried out in an AlphaSC cycler type (Analytik Jena AG, Jena, Germany), and the pyrosequencing was performed using PyroMark Q24 Advanced reagents (Qiagen), whereas the acquired sequences were analyzed with PyroMark Q24 2.0.6 software (Biotage AB, Uppsala, Sweden) to evaluate the sequence quality. Genotyping was performed in a blinded fashion without knowledge of the patients' clinical data, and 10% of the samples were genotyped in duplicate to monitor genotyping quality.

Regarding the platelet function assay, the blood samples were drawn using a 21-gauge needle to collect the samples, which were promptly transferred to plastic tubes containing 3.2% buffered sodium citrate solution. The ratio of blood to citrate was maintained at 9:1. The samples were then gently mixed with the anticoagulant. After acquiring the sample, hematocrit and platelet counts were assessed and subsequently validated through the evaluation of peripheral blood smears. Blood samples were kept at ambient temperature, and thereafter, all samples were subjected to testing with COL/EPI cartridges using the PFA-100® system (Dade Behring, Deerfield, IL) within a maximum of four hours, following the guidelines provided by the manufacturer. Every individual test cartridge was equipped with a collagen-coated membrane with an opening measuring 147 µm in diameter. The membrane was further covered with a 10 μg epinephrine (COL/EPI cartridge), which functions as a platelet agonist. A volume of 0.8 mL of citrate-anticoagulated whole blood was drawn through the aperture using negative pressure, resulting in the generation of high shear stress and mimicking the circumstances found in actual blood vessels. The activation of platelets and the subsequent development of a primary clot were observed during the incubation of whole blood with the agonist. The occurrence in question signified the ultimate stage in the process of quantifying primary hemostasis and was documented as CT, denoting the duration in seconds required for the system to generate a platelet clot in response to each agonist. Coagulation and epithelialization closure times (COL/EPI CTs) were assessed for each sample, with the highest CT recorded by the PFA-100 being 300 seconds. Samples with a CT of >300 seconds were classified as "non-closure" and were subsequently eliminated from the study, resulting in the exclusion of all samples containing clots.

The PFA-100 cartridge system is designed for in vitro evaluation of primary hemostasis related to platelets. It is particularly sensitive to various preanalytical factors, including hemostatic defects, medication effects, platelet deficiencies, and hematocrit disturbances. These factors were accounted for in the analysis to ensure more reliable and significant results. COL/EPI CT values were determined. Blood group and complete blood count tests (Cell-Dyn 3,700 hematology analyzer, Abbott, IL) were also performed. Microscopic examination of peripheral blood smears corroborated the platelet count.

Statistical analyses were performed using Statistical Package for the Social Sciences (SPSS) software version 22.0 (IBM, Armonk, NY). Quantitative variables were expressed as mean ± standard deviation (SD) and/or median ± interquartile range (IQR) (25th-75th percentile), and categorical variables were presented as absolute frequencies and percentages (%). Pearson's chi-square and Fisher's exact tests were used for frequency comparison, and the associations were expressed as odds ratios (OR) with 95% confidence intervals (95% CI). The Hardy-Weinberg equilibrium (HWE) was confirmed by using Pearson's chi-square goodness of fit. For the quantitative variables, distribution normality was checked using the Kolmogorov-Smirnov test, and the one-way analysis of variance (ANOVA) and the Tukey post hoc test were used for comparisons, whereas the parametric Pearson's r and the nonparametric Spearman's rho coefficients were used for correlations, as appropriate. The level of statistical significance was set at p-value < 0.05.

## Results

The frequency distributions of allele and genotype frequencies in women in our population with respect to the GpIa-C807T and GpIIIa-PlA1/PlA2 genetic polymorphisms are presented in Table 1.

**Table 1:** GpIa-C807T and GpIIIa-PlA1.PlA2 allele and genotype frequencies in women with recurrent miscarriages

	GpIa-C807T	Number	(%)	GpIIIa-PlA1/PlA2	Number	%
Alleles	GpIa-807T	150	38.3	GpIIIa-PlA2	77	19.6
	GpIa-807C	242	61.7	GpIIIa-PlA1	315	80.4
Genotypes	GpIa-807TT	28	14.3	GpIIIa-PlA2PlA2	11	5.6
	GpIa-807CT	94	48.0	GpIIIa-PlA1PlA2	55	28.1
	GpIa-807CC	74	37.8	GpIIIa-PlA1PlA1	130	66.3

The frequency of GpIa-807C and GpIa-807T alleles was 61.7% and 38.3%, respectively. Twenty-eight (14.3%) women were homozygous for the GpIa-807T allele (genotype: GpIa-807TT), and 94 (48%) were heterozygotes (genotype: GpIa-807CT). Furthermore, the frequency of GpIIIa-PlA1 and GpIIIa-PlA2 alleles was 80.4% and 19.6%, respectively. Eleven (5.6%) women were homozygous for the GpIIIa-PlA2 allele (genotype: GpIIIa-PlA2PlA2), and 55 (28.1%) were heterozygotes (genotype: GpIIIa-PlA1PlA2).

The genotype frequency distributions of the studied variants were in agreement with the HWE (GpIa-C807T: p=0.714, GpIIIa-PlA1/PlA2: p=0.185).

The frequency distributions of the combined genotypes in women with recurrent miscarriages are presented in Table 2.

**Table 2:** Distribution of the combined GpIa-C807T and GpIIIa-PlA1/PlA2 genotypes in women with recurrent miscarriages

Combined GpIa-C807T and GpIIIa-PlA1/PlA2 genotypes	Number	%
TT/PlA2PlA2	0	0
TT/PlA1PlA2	10	5.1
TT/PlA1PlA1	18	9.2
CT/PlA2PlA2	6	3.1
CT/PlA1PlA2	23	11.7
CT/PlA1PlA1	65	33.2
CC/PlA2PlA2	5	2.6
CC/PlA1PlA2	22	11.2
CC/PlA1PlA1	47	24
GpIa-807T and GpIIIa-PlA2 combined carriers (genotypes: TT/PlA1PlA2, CT/PlA2PlA2, CT/PlA1PlA2)	39	19.9
GpIa-807T single carriers (genotypes: TT/PlA1PlA1, CT/PlA1PlA1)	83	42.3
GpIIIa-PlA2 single carriers (genotypes: CC/PlA2PlA2, CC/PlA1PlA2)	27	13.8

**Figure 1:**
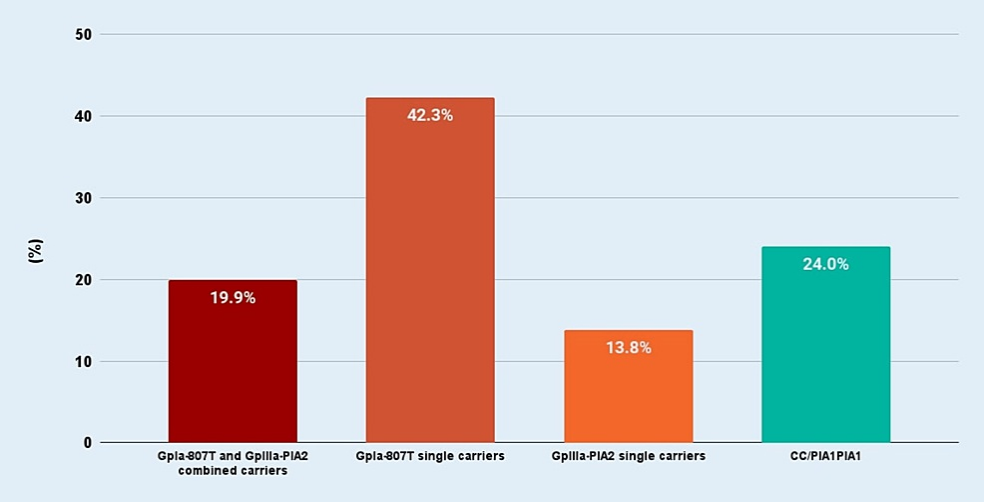
Distribution of women with recurrent miscarriages according to the combined GpIa-807T and GpIIIa-PlA2 alleles carriage

In our sample, 39 (19.9%) women were combined GpIa-807T and GpIIIa-PlA2 carriers, 10 (5.1%) with TT/PlA1PlA2, six (3.1%) with CT/PlA2PlA2, and 23 (11.7%) with CT/PlA1PlA2. Moreover, 83 (42.3%) women were single GpIa-807T carriers (GpIa-807T carriers and PlA1PlA1 homozygotes), 18 (9.2%) with the TT/PlA1PlA1 genotype and 65 (33.2%) with the CT/PlA1PlA1 genotype, whereas 27 (13.8%) patients were single GpIIIa-PlA2 carriers (GpIIIa-PlA2 carriers and CC homozygotes) (genotypes: five or 2.6% with CC/PlA2PlA2 and 22 or 11.2% with CCPlA1PlA2). The remaining 47 (24%) subjects had the GpIa-807CC/GpIIIa-PlA1PlA1 genotype (Table 1, Figure 1).

In the total population of women with recurrent miscarriages, the COL/EPI CT ranged from 70 to 160 seconds, with a mean of 120 seconds (± 22.2 seconds) and a median of 122 seconds (IQR: 102.3-138 seconds) (Table 3).

**Table 3:** Distribution of the COL/EPI CT in the total population of women with recurrent miscarriages COL/EPI CT: collagen/epinephrine closure time, SD: standard deviation

COL/EPI CT (seconds)		Total
Mean		120
SD		22.2
Median		122
Minimum		70
Maximum		160
Percentiles	10th	90
	25th	102.3
	75th	138
	90th	148.3
Normality test (p-value)	Kolmogorov-Smirnov	0.012

**Figure 2:**
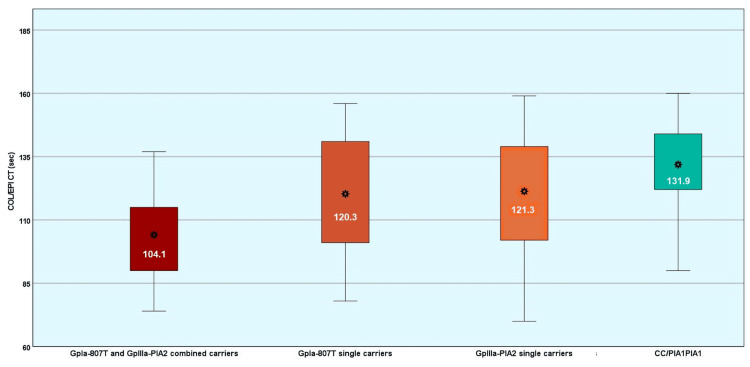
Boxplot diagram of the distribution of the COL/EPI CT according to the presence of the GpIa-807T and GpIIIa-PlA2 alleles COL/EPI CT: collagen/epinephrine closure time

The COL/EPI CT was statistically significantly different among the combined carriers, the single carriers, and the non-carriers of the GpIa-807T and the GpIIIa-PlA2 alleles (p<0.001) (Table 4, Figure 2).

**Table 4:** Distribution of the COL/EPI CT in GpIa-807T and GpIIIa-PlA2 combined carriers, single polymorphism carriers, and women with the CC/PlA1PlA1 genotype COL/EPI CT: collagen/epinephrine closure time, SD: standard deviation

COL/EPI CT (seconds)		GpIa-807T and GpIIIa-PlA2 combined carriers	GpIa-807T single carriers	GpIIIa-PlA2 single carriers	CC/PlA1PlA1
Mean		104.1	120.3	121.3	131.9
SD		19.7	20.9	23.7	17.5
Median		103	121	125	132
Minimum		74	78	70	90
Maximum		156	156	159	160
Percentiles	25th	90	101	102	122
	75th	115	141	139	144
Normality test (p-value)	Kolmogorov-Smirnov	0.200	0.050	0.200	0.200

In comparison with the CC/PlA1PlA1 group that had the most prolonged CT (mean: 131.9 ± 17.5 seconds), the COL/EPI CT was statistically significantly shorter for the GpIa-807T single carriers (mean: 120.3 ± 20.9 seconds) (p=0.011) (absolute difference: -11.6 seconds, 95% CI: -21.2 to -2.0 seconds; relative difference: -9%, 95% CI: -16% to -2%). In addition, single carriers of the GpIIIa-PlA2 allele displayed a trend for shorter COL/EPI CT (mean: 121.3 ± 23.7 seconds) (p=0.141) (absolute difference: -10.6 seconds, relative difference: -8%). Women who were combined carriers of the GpIa-807T and the GpIIIa-PlA2 alleles exhibited the shortest COL/EPI CT (mean: 104.1 ± 19.7 seconds) compared with the CC/PlA1PlA1 group (absolute difference: -27.7 seconds, 95% CI: -39.1 to -16.3 seconds; relative difference: -21%, 95% CI: -30% to -12%) (p<0.001). Combined GpIa-807T and GpIIIa-PlA2 carriers displayed shorter COL/EPI CT in comparison with the GpIIIa-PlA2 single carriers (absolute difference: -17.2 seconds, 95% CI: -30.4 to -4.0 seconds; relative difference: -13%, 95% CI: -23% to -3%) (p=0.005) and in comparison with the GpIa-807T single carriers (absolute difference: -16.2 seconds, 95% CI: -26.4 to -6.0 seconds; relative difference: -12%, 95% CI: -20% to -4%) (p<0.001). The COL/EPI CT did not differ between the single carriers of the GpIa-807T and the GpIIIa-PlA2 carriers (p=0.996) (Table 5, Figure 2).

**Table 5:** Comparisons of the COL/EPI CTs among combined carriers, single carriers, and non-carriers of the GpIa-807T and GpIIIa-PlA2 alleles COL/EPI CT: collagen/epinephrine closure time, CI: confidence interval

Groups	Difference (seconds)	95% CI (seconds)	p-value
GpIa-807T single carriers versus CC/PlA1PlA1	-11.6	-21.2 to -2.0	0.011
GpIIIa-PlA2 single carriers versus CC/PlA1PlA1	-10.6	-23.4 to 2.2	0.141
GpIa-807T and GpIIIa-PlA2 combined carriers versus CC/PlA1PlA1	-27.7	-39.1 to -16.3	<0.001
GpIa-807T and GpIIIa-PlA2 combined carriers versus GpIIIa-PlA2 single carriers	-17.2	-30.4 to -4.0	0.005
GpIa-807T and GpIIIa-PlA2 combined carriers versus GpIa-807T single carriers	-16.2	-26.4 to -6.0	<0.001
GpIa-807T versus GpIIIa-PlA2 single carriers	-1.0	-12.7 to 10.7	0.996

The proportion of women with COL/EPI CT < 122 seconds (median of the total distribution) was significantly higher in the GpIa-807T single carriers (42/83 (50.6%)) and among GpIIIa-PlA2 single carriers (12/27 (44.4%)) compared with non-carriers of the GpIa-807T and GpIIIa-PlA2 alleles (11/47 (23.4%)) (OR=3.4, 95% CI: 1.5 to 7.5, p=0.002, and OR=2.6, 95% CI: 1.0 to 7.2, p=0.053, respectively). This association was strongest for the combined carriers subgroup: 32/39 (82.1%) had COL/EPI CT < 122 seconds with OR of 15.0 (95% CI: 5.2 to 43.2, p<0.001), whereas the OR of 35.5 (95% CI: 4.4 to 284.5, p<0.001) when comparing the proportion of patients with COL/EPI CT < 100 seconds between these subgroups (combined carriers: 17/39 (43.6%) versus double homozygotes: 1/47 (2.1%)) (Table 6).

**Table 6:** Associations of the GpIa-807T and GpIIIa-PlA2 alleles carriers with COL/EPI CT below the median (122 seconds) or shorter than 100 seconds COL/EPI CT: collagen/epinephrine closure time, OR: odds ratio, CI: confidence interval

Genotypes	COL/EPI CT (seconds)		Total	OR	95% CI	p-value
	<122	≥122				
GpIa-807T and GpIIIa-PlA2 combined carriers	32 (82.1%)	7 (17.9%)	39	15.0	5.2 to 43.2	<0.001
GpIa-807T single carriers	42 (50.6%)	41 (49.4%)	83	3.4	1.5 to 7.5	0.002
GpIIIa-PlA2 single carriers	12 (44.4%)	15 (55.6%)	27	2.6	1.0 to 7.2	0.053
CC/PlA1PlA1	11 (23.4%)	36 (76.6%)	47	-	-	-
	<100	≥ 100				
GpIa-807T and GpIIIa-PlA2 combined carriers	17 (43.6%)	22 (56.4%)	39	35.5	4.4 to 284.5	<0.001
CC/PlA1PlA1	1 (2.1%)	46 (97.9%)	47	-	-	-

The age of our sample with recurrent miscarriages ranged from 24 to 40 years (mean: 32.8 ± 4.1 years, median: 32 years, IQR: 30-36 years). There was no statistically significant correlation between the COL/EPI CT and maternal age in the whole population (rho=-0.077, p=0.282), in the GpIa-807T carriers (r=-0.063, p=0.571), in the GpIIIa-PlA2 carriers (r=-0.364, p=0.062), or among the combined GpIa-807T and GpIIIa-PlA2 carriers (r=0.098, p=0.553).

## Discussion

This cross-sectional study revealed significant associations between the platelet function in women with recurrent miscarriages determined via PFA-100 and the genetic polymorphisms GpIa-C807T and GpIIIa-PlA1/PlA2 of the platelet glycoprotein receptors Ia and IIIa. 

This was a secondary analysis of a group of 222 women with a history of repeated unexplained spontaneous abortions, genotyped by pyrosequencing for the presence of the GpIa-C807T and GpIIIa-PlA1/PlA2 genetic polymorphisms. The analysis revealed statistically significant associations between the GpIa-807T and GpIIIa-PlA2 polymorphic alleles and the risk of miscarriage [11]. Under a dominant genetic model [13], the carriers of the GpIa-807T and GpIIIa-PlA2 alleles had increased risks of miscarriage (OR=3.36 and OR=2.58, respectively), compared with the common alleles' homozygotes (GpIa-807CC and GpIIIa-PlA1PlA1, respectively) [11].

One hundred ninety-six women of this cohort were further investigated regarding platelet function by PFA-100, and COL/EPI CTs were determined. Our results indicated statistically significant associations between the presence of the GpIa-807T and GpIIIa-PlA2 risk alleles and shorter COL/EPI CTs.

Specifically, in order to reveal the individual effect of each risk allele on platelet function, COL/EPI CTs were determined in the group of women who carried the GpIa-807T, but not the GpIIIa-PlA2, allele (single GpIa-807T carriers, genotypes TT/PlA1PlA1, CT/PlA1PlA1) and in the group of women who were carriers of the GpIIIa-PlA2, but not the GpIa-807T, alleles (single GpIIIa-PlA2 carriers, genotypes CC/PlA2PlA2, CC/PlA1PlA2) and were compared with those who were double homozygotes CC/PlA1PlA1 who displayed the most prolonged COL/EPI CT and were used as a reference.

Our results showed that, compared with the CC/PlA1PlA1 genotype, women who were single carriers of the GpIa-807T allele had statistically significantly shorter COL/EPI CT, and single carriers of the GpIIIa-PlA2 allele also exhibited shorter COL/EPI CT, although it did not reach statistical significance. The mean COL/EPI CT was 9% and 8% shorter in the GpIa-807T and the GpIIIa-PlA2 single carriers groups, respectively.

Moreover, the COL/EPI CT was even further shortened among the patients who were combined carriers of the risk alleles GpIa-807T and GpIIIa-PlA2, with a mean COL/EPI CT 21% shorter than that in the CC/PlA1PlA1 genotype. Interestingly, this finding was in accordance with the observation of an elevated risk of pregnancy loss in the primary analysis [11], for combined carriers of both polymorphic alleles (GpIa-807T and GpIIIa-PlA2), compared with those with the CC/PlA1PlA1 genotype, indicating an interaction between GpIa-807T and GpIIIa-PlA2 alleles on the risk of fetal loss, which was also evident in platelet function.

Furthermore, we compared the frequency of women with COL/EPI CTs shorter than the median value of the overall COL/EPI CT distribution (122 seconds) among genotypes. We found that 60.7% of the GpIa-807T allele carriers and 66.7% of the GpIIIa-PlA2 allele carriers had a COL/EPI CT < 122 seconds, compared with 31.1% and 40.8% among non-carriers, respectively (OR = 3.4 and OR = 2.9, respectively), whereas combined carriers of the risk variants GpIa-807T and GpIIIa-PlA2 were 15-fold more likely to exhibit a COL/EPI CT below the median than those with the CC/PlA1PlA1 genotype. The above association was more pronounced after taking as a reference level the COL/EPI CT value of 100 seconds, which is a critically short value strongly associated with increased platelet reactivity [14]. It was found that the combined carriers of the GpIa-807T and GpIIIa-PlA2 alleles had more than 35-fold higher odds of COL/EPI CT < 100 seconds, in comparison with the double homozygotes CC/PlA1PlA1.

The GpIa/GpIIa complex (integrin alpha2beta1) is a major platelet receptor that binds to collagen and plays an important role in platelet adhesion and activation. The genetic polymorphism GpIa-C807T, although causing a synonymous mutation in the glycoprotein Ia gene, has been shown to be associated with a fourfold variation in the expression of GpIa/GpIIa surface platelet receptors. In particular, the GpIa-807T variant has been associated with increased receptor concentration, whereas the GpIa-807C variant with low expression. A number of clinical studies have reported positive associations of the GpIa-807T allele with a higher risk of arterial thrombosis and increased platelet aggregation [15,16].

The GpIIb-IIIa complex (integrin alphaIIbbeta3) is a platelet membrane receptor for fibrinogen and von Willebrand factor. The GpIIIa-PlA1/PlA2 genetic polymorphism results in the alternation of the amino acids leucine (PlA1) and proline (PlA2) at position 33 of the glycoprotein IIIa peptide chain modifying the affinity and avidity of the receptor for ligands and enhancing outside-in signaling. Clinical evidence has also suggested that the GpIIIa-PlA2 variant is a risk allele for myocardial infarction and ischemic stroke [17,18].

The PFA-100 is an in vitro analyzer system of platelet function that can accurately measure platelet-related primary hemostasis. In a continuous range of values, a short CT is indicative of an increased risk of thrombosis, whereas a prolonged CT has been associated with increased bleeding risk. The PFA-100 has been extensively evaluated in a variety of physiological and pathological conditions in more than 1,000 publications, including platelet function defects such as the von Willebrand disease, surgical bleeding risk, as well as stroke and myocardial ischemia, and venous thrombosis risk [19,20]. PFA-100 has also been used to assess platelet function in normal pregnant women and in high-risk pregnancies, such as those complicated by preeclampsia and gestational diabetes mellitus [21-23]. Additionally, two recent publications reported prolonged COL/EPI CT in healthy preterm neonates [24] and in those born with fetal growth restriction [25]. To our knowledge, this was the first study in the literature examining platelet function using PFA-100 in a population of women with recurrent miscarriages.

Primary genetic analysis of the cohort showed statistically significantly increased risks of miscarriage for women carrying the GpIa-807T and GpIIIa-PlA2 alleles [11]. Secondary analysis indicated that women carrying the risk alleles also had increased platelet reactivity, expressed by statistically significantly shorter COL/EPI CTs. Furthermore, genetic analysis revealed a significant synergistic interaction between the coexistence of the GpIa-807T and GpIIIa-PlA2 alleles resulting in further increased risk [11]. It is of particular interest that the study via PFA-100 demonstrated strongly impaired platelet function for women with combined carriage of GpIa-807T and GpIIIa-PlA2 alleles, expressed with greatly shortened COL/EPI CT time. In vitro studies have reported increased GpIa-IIa receptor density in carriers of the GpIa-807T allele and enhanced affinity and avidity of the GpIIb-IIIa receptor attributed to the GpIIIa-PlA2 allele, suggesting plausible biological mechanisms explaining our findings [11,15-18]. Two decades ago, it was first observed that a young individual with double homozygosity of the prothrombotic alleles GpIa-807T and GpIIIa-PlA2 showed shortened PFA-100 CT and an increased platelet aggregation response to collagen [26]. In another study of 286 healthy subjects, an increased aggregation response to collagen was also found in platelets of healthy subjects who carried the risk alleles [27].

In this study, we used the PFA-100 as a point-of-care diagnostic tool and found that the GpIa-807T and the GpIIIa-PlA2 alleles are associated with enhanced platelet function expressed by significantly shorter COL/EPI CTs resulting in hypercoagulability due to increased platelet activation. These results were obtained by examining a strictly selected sample of women with miscarriages who had been previously screened for the presence of known risk factors for early fetal loss. Besides the relatively small sample size, another limitation of the study could be the non-assessment of the von Willebrand factor levels in the blood samples of our subjects [14].

The results of the present study suggest that the detrimental effects of the prothrombotic GpIa-807T and GpIIIa-PlA2 alleles in miscarriages probably occur through the impairment of maternal platelet function resulting in hypercoagulability during the first crucial weeks of gestation. Platelets with high aggregability can divert the delicate balance of hemostasis toward thrombosis with devastating consequences for the progression and development of pregnancy, yet evidence supporting the role of platelet function in promoting miscarriage is insufficient. Increased platelet aggregation in women with unexplained recurrent miscarriages has been reported by a few small studies [28], and in addition, sticky platelet syndrome, an inherited thrombocytopathy characterized by platelet hyperaggregation and hypercoagulation, also appears to be a risk factor for fetal loss [29]. Our findings provide further evidence for the pathophysiology and importance of platelet-associated genetic thrombophilia in the pathogenesis of recurrent miscarriages as well as the potential value of platelet function assessment in the diagnostic approach to this disorder.

## Conclusions

We identified significant associations of the GpIa-C807T and the GpIIIa-PlA1/PlA2 genetic polymorphisms with platelet function evaluated via PFA-100 in women with unexplained recurrent miscarriages. Carriers of the susceptibility alleles for spontaneous miscarriages GpIa-807T and GpIIIa-PlA2 exhibited shorter COL/EPI CT, whereas the combined carriers of the risk variants demonstrated a more pronouncedly overresponsive platelet phenotype. Our findings further elucidate the role of platelet-related inherited thrombophilia in the etiopathogenesis of recurrent miscarriages and suggest the analysis of platelet function as a diagnostic tool in the evaluation of this disorder.
